# Sitagliptin Potentiates the Anticancer Activity of Doxorubicin Through ROS-Driven Apoptosis and MMP/TIMP Regulation in HeLa Cells

**DOI:** 10.3390/pharmaceutics18010038

**Published:** 2025-12-26

**Authors:** Aşkın Evren Güler, Mehmet Cudi Tuncer, İlhan Özdemir

**Affiliations:** 1 Department of Gynecology and Obstetrics, Aşkın Evren Güler Medical Clinic, Ankara 06560, Turkey; askinevrenguler@yahoo.com; 2 Department of Anatomy, Faculty of Medicine, Dicle University, Diyarbakır 21280, Turkey; 3 Department of Histology Embryology, Faculty of Medicine, Kahramanmaraş Sütçü İmam University, Kahramanmaraş 46100, Turkey; ilhanozdemir25@yandex.com

**Keywords:** sitagliptin, doxorubicin, cervical cancer, apoptosis, PI3K/Akt pathway

## Abstract

**Background/Objectives**: Cervical cancer remains a major global health challenge, and treatment resistance limits the long-term success of chemotherapy. Drug repurposing strategies offer new opportunities for improving therapeutic outcomes by combining existing agents with established chemotherapeutics. Sitagliptin, a DPP-4 inhibitor commonly used in type 2 diabetes, has recently gained attention for its potential anticancer effects. This study aimed to investigate the cytotoxic, apoptotic, and anti-metastatic effects of sitagliptin and doxorubicin, individually and in combination, on human cervical cancer cells (HeLa), and to determine whether their combined use exerts a synergistic anticancer effect. **Methods**: HeLa cells were treated for 48 h with increasing concentrations of sitagliptin, doxorubicin, or their combination. Cell viability was assessed using the MTT assay. Apoptosis was evaluated by Annexin V-FITC/PI staining and caspase-8/9 activity assays. Synergy was quantified using the Chou–Talalay method, and Combination Index (CI) values were used to determine synergistic interactions. Intracellular ROS levels were measured using the DCFDA assay. Migration and invasion capacities were analyzed using wound healing and Transwell assays. MMP-1, MMP-2, TIMP-1, and TIMP-2 levels were quantified via ELISA with normalization to viable cell counts. Gene expression levels of PI3K/Akt and MAPK/ERK pathway components were measured by qRT-PCR. Bioinformatic analyses (STRING, GeneMANIA, GO, KEGG) were performed to identify common molecular targets and enriched pathways affected by both agents. **Results**: The combination of sitagliptin and doxorubicin significantly reduced cell viability and demonstrated a synergistic interaction (CI < 1). Combined treatment induced a marked increase in ROS production and significantly elevated apoptosis rates compared to monotherapies. Caspase-8 and caspase-9 activities were also higher in the combination group. Migration and invasion assays revealed substantial suppression of cell motility and invasive capacity. After normalization to viable cell numbers, MMP and TIMP reductions remained significant, confirming true biological inhibition rather than cell-death–related artifacts. qRT-PCR analyses showed downregulation of Akt and ERK expression, indicating suppression of key survival and proliferation pathways. Bioinformatic analyses supported these findings by highlighting enrichment in apoptotic, oxidative stress, and metastasis-related pathways. **Conclusions**: Sitagliptin enhances the anticancer efficacy of doxorubicin by amplifying ROS-mediated apoptosis, inhibiting migration and invasion, and modulating PI3K/Akt and MAPK/ERK signaling in cervical cancer cells. The combination exhibits a clear synergistic effect and demonstrates strong potential as a supportive therapeutic strategy. These findings warrant further in vivo and clinical-level investigations to evaluate the translational applicability of sitagliptin in cervical cancer therapy.

## 1. Introduction

Type 2 diabetes mellitus (T2DM) is a chronic metabolic disorder characterized by insulin resistance and progressive β-cell dysfunction [1]. Beyond its well-known metabolic complications, accumulating epidemiological evidence has identified T2DM as an important risk factor for several malignancies [2]. Indeed, individuals with diabetes demonstrate higher incidences of pancreatic, liver, colorectal, endometrial, and breast cancers [1,2]. Mechanistically, diabetes-associated hyperglycemia, hyperinsulinemia, and chronic low-grade inflammation promote tumor cell proliferation, angiogenesis, epithelial–mesenchymal transition, and metastasis, thereby contributing to cancer initiation and progression [2].

Growing interest has focused on how antidiabetic medications influence cancer biology. Among these, dipeptidyl-peptidase-4 (DPP-4) inhibitors have attracted attention due to their potential extraglycemic effects. Although these agents primarily enhance incretin signaling and regulate glucose-dependent insulin secretion [3], DPP-4 also functions as a cell-surface adhesion molecule involved in immune regulation, cell migration, and extracellular matrix remodeling [4]. Consequently, DPP-4 inhibition has been suggested to modulate tumor behavior through mechanisms related to proliferation, adhesion, apoptosis, and metastasis [3,4]. Notably, DPP-4 overexpression in several tumor types has been associated with enhanced metastatic potential, making sitagliptin—a widely prescribed DPP-4 inhibitor—a promising candidate for anticancer drug-repurposing research [5,6,7].

Recent evidence indicates that the role of DPP-4 and its pharmacological inhibition in female malignancies is highly context-dependent. A comprehensive systematic review integrating in vitro, in vivo, and in silico studies reported that DPP-4 expression and the effects of DPP-4 inhibitors vary substantially across breast, ovarian, cervical, and endometrial cancers, with both tumor-promoting and tumor-suppressive roles described depending on cancer type and molecular context [8]. Notably, in cervical cancer models, DPP-4 expression has been implicated in the regulation of cell migration, and sitagliptin has been shown to significantly reduce cell adhesion in vitro, even in cell lines with minimal CD26 expression [8,9]. These findings highlight the need for mechanistic studies that clarify how DPP-4 inhibition modulates apoptosis, migration, and invasion in cervical cancer cells, particularly in combination with conventional chemotherapeutic agents.

Cervical cancer remains one of the most prevalent gynecological malignancies worldwide. While persistent infection with high-risk human papillomavirus is the primary etiological factor, metabolic disorders such as obesity and diabetes have been identified as important co-factors influencing cervical carcinogenesis, treatment response, and prognosis [10,11,12]. Standard chemotherapeutic agents, including doxorubicin, are commonly used in advanced or recurrent cervical cancer; however, their effectiveness is often limited by systemic toxicity, chemoresistance, and inadequate tumor selectivity [13]. Therefore, the identification of agents capable of enhancing doxorubicin efficacy while minimizing adverse effects is of considerable therapeutic interest.

In this context, the present study aimed to investigate the anticancer effects of sitagliptin alone and in combination with doxorubicin in HeLa cells. Specific focus was given to the modulation of cell viability, apoptosis, migration, invasion, and extracellular matrix–related factors such as metalloproteinases (MMPs) and tissue inhibitors of metalloproteinases (TIMPs). Additionally, alterations in key signaling pathways associated with cancer progression were explored [14,15]. Although the combined use of sitagliptin and doxorubicin has not yet been established in clinical practice, elucidating their potential synergistic interaction in vitro may provide an important scientific basis for future preclinical investigations and the development of novel adjuvant therapeutic strategies.

## 2. Materials and Methods

### 2.1. Cell Culture

The human cervical adenocarcinoma cell line HeLa (ATCC^®^ CCL-2™) was used in all experiments. Cells were maintained in Dulbecco’s Modified Eagle Medium (DMEM; Gibco, Thermo Fisher Scientific, Waltham, MA, USA) supplemented with 10% fetal bovine serum (FBS) and 1% penicillin–streptomycin. Cultures were incubated at 37 °C in a humidified atmosphere containing 5% CO_2_. When cell confluence reached approximately 80%, cells were detached using 0.25% trypsin–EDTA, centrifuged, and reseeded for subsequent experiments. All assays were performed using cells in the logarithmic growth phase.

### 2.2. Treatment Groups

Four experimental groups were established:

1. Control group: Untreated HeLa cells.
2. Sitagliptin group: Cells were treated with sitagliptin at 5, 10, 20, 40, 80, and 100 µM for 48 h.
3. Doxorubicin group: Cells were treated with doxorubicin at 0.5, 1, 5, 10, 20, and 50 µM for 48 h.
4. Sitagliptin + Doxorubicin group: Cells were treated with both agents simultaneously at a fixed 1:1 ratio based on their respective IC_50_ values, as calculated from individual dose–response curves.

### 2.3. Cell Viability Analysis (MTT Assay)

Cell viability was assessed using the MTT (3-[4,5-dimethylthiazol-2-yl]-2,5-diphenyltetrazolium bromide) assay. HeLa cells were seeded into 96-well plates at a density of 1 × 10^4^ cells per well and allowed to adhere overnight. Following treatment, the drug-containing medium was completely removed and cells were washed with PBS to eliminate any residual sitagliptin or doxorubicin prior to MTT incubation. Then, 20 µL of MTT solution (0.5 mg/mL) was added to each well and incubated for 4 h at 37 °C. The resulting formazan crystals were dissolved in 100 µL of fresh DMSO, and absorbance was measured at 570 nm using a microplate reader (BioTek ELx800, BioTek Instruments, Winooski, VT, USA). Since absorbance readings were taken from DMSO-dissolved formazan only, without residual drug present, interference from the intrinsic color of doxorubicin was avoided.

Cell viability was expressed as a percentage relative to the untreated control. IC_50_ values for sitagliptin and doxorubicin were determined from non-linear dose–response curves using a four-parameter logistic model in GraphPad Prism 9.0 (GraphPad Prism, version 9.0; GraphPad Software, San Diego, CA, USA). For combination studies, the concentrations corresponding to the calculated IC_50_ values of each agent were used following the Chou–Talalay method.

### 2.4. Synergism Analysis Using the Chou–Talalay Method

The interaction between sitagliptin and doxorubicin was evaluated using the Chou–Talalay CI method. First, individual dose–response curves for each drug were generated by treating HeLa cells with sitagliptin and doxorubicin at 0.25×, 0.5×, 1×, 2×, and 4× their IC_50_ concentrations, and the corresponding fraction affected (FA) values were calculated based on cell viability.

For the combination studies, sitagliptin and doxorubicin were administered simultaneously at a fixed 1:1 ratio, determined according to their respective IC_50_ values. The same graded dose levels (0.25×–4× IC_50_) were applied to the combination to maintain the fixed-ratio design, as required by the Chou–Talalay method. FA values obtained from all single-agent and combination treatments were imported into CompuSyn version 1.0 (ComboSyn Inc., Paramus, NJ, USA).

CompuSyn automatically generated CI and Dose Reduction Index (DRI) values for multiple effect levels (FA = 0.25, 0.50, 0.75, and 0.90). CI values were interpreted as follows: CI < 1 indicates synergism, CI = 1 indicates an additive effect, and CI > 1 indicates antagonism. In addition, isobologram plots and CI–FA (Fa–CI) curves were generated to visualize the pharmacological interaction between the two agents and to validate synergy across different effect levels.

### 2.5. Reactive Oxygen Species (ROS) Measurement

Intracellular ROS levels were quantified using the 2′,7′-dichlorofluorescin diacetate (DCFDA; Sigma-Aldrich, St. Louis, MO, USA) fluorescent probe. HeLa cells were seeded into 96-well plates at a density of 1 × 10^4^ cells per well and allowed to adhere overnight. Following treatment, cells were washed with PBS and incubated with 10 µM DCFDA prepared in serum-free medium for 30 min at 37 °C in the dark. After incubation, excess dye was removed by washing the wells with PBS. Fluorescence intensity was measured using a microplate reader at λ_ex = 485 nm and λ_em = 530 nm. A hydrogen peroxide (H_2_O_2_)-treated group served as a positive control. ROS levels were normalized to the untreated control group and expressed as relative fluorescence units (RFU).

### 2.6. Apoptosis Analysis

Apoptosis was evaluated using Annexin V-FITC/Propidium Iodide (PI) dual staining followed by flow cytometry. HeLa cells were seeded into 6-well plates (2 × 10^5^ cells/well) and treated under the specified experimental conditions. After treatment, both floating and adherent cells were collected, washed twice with cold PBS, and resuspended in 1× Annexin binding buffer. Cells were stained with 5 µL Annexin V-FITC and 5 µL PI (BD Biosciences, San Jose, CA, USA) and incubated for 15 min at room temperature in the dark. Samples were analyzed immediately using a BD Accuri C6 flow cytometer, and at least 10,000 events per sample were recorded. The proportions of viable, early apoptotic, late apoptotic, and necrotic cells were determined based on quadrant gating.

For flow cytometric analysis, quadrant gating was performed based on Annexin V–FITC and propidium iodide (PI) fluorescence. Gates defining viable (Annexin V^−^/PI^−^), early apoptotic (Annexin V^+^/PI^−^), late apoptotic (Annexin V^+^/PI^+^), and necrotic (Annexin V^−^/PI^+^) cell populations were established using untreated control cells. The same quadrant thresholds were then applied consistently to all experimental groups to ensure accurate comparison of apoptotic populations.

Caspase-8 and caspase-9 activities were assessed using commercial colorimetric assay kits (Abcam, Cambridge, UK) according to the manufacturer’s instructions. Briefly, cell lysates were prepared after treatment, and substrate cleavage was monitored by measuring absorbance at 405 nm on a microplate reader. Enzyme activity was expressed as fold change relative to the untreated control, calculated from changes in optical density (OD).

#### NucBlue Nuclear Staining for Morphological Assessment of Apoptosis

HeLa cells were seeded on sterile glass coverslips placed in 24-well plates at a density of 1 × 10^5^ cells per well and allowed to adhere overnight. Following treatment with sitagliptin (IC_50_ = 52.4 µM), doxorubicin (IC_50_ = 4.18 µM), or their combination for 48 h, cells were washed twice with PBS. Cells were stained with the NucBlue Live ReadyProbes reagent (Thermo Fisher Scientific, Waltham, MA, USA) according to the manufacturer’s instructions and incubated for 20 min at room temperature in the dark. After staining, coverslips were mounted with Fluoromount medium.

Nuclear morphology was imaged using a fluorescence microscope (DAPI filter, 200× magnification). Chromatin condensation and nuclear fragmentation were assessed qualitatively, while fluorescence intensity was quantified using ImageJ (version 1.53; National Institutes of Health, Bethesda, MD, USA) from ≥90 nuclei per group across three independent experiments. Fluorescence intensity analysis was performed in ImageJ. For each sample, individual nuclei were manually outlined as regions of interest, background fluorescence was subtracted, and the mean gray value of each nucleus was recorded. A minimum of 50 nuclei per treatment group were analyzed, and final fluorescence intensity values (AU) represent the average of all measured nuclei.

These nuclear morphology images and the corresponding quantitative fluorescence measurements were generated using the ImageJ analysis workflow described above and are presented in Section 3.2 as representative and quantitative assessments of apoptosis-related nuclear changes.

### 2.7. Wound Healing Migration Assay

The wound healing assay was performed to assess the two-dimensional migratory capacity of HeLa cells. Cells were seeded into 6-well plates at a density of 3 × 10^5^ cells per well and cultured until a confluent monolayer was formed. A straight and uniform scratch was created across the monolayer using a sterile 200 µL pipette tip, guided perpendicular to the well surface to ensure consistent scratch width. Detached cells and debris were removed by gently washing twice with PBS.

Cells were then incubated in serum-reduced medium (DMEM + 1% FBS) containing sitagliptin, doxorubicin, or their combination for 48 h to minimize proliferation-related effects and isolate migration-driven wound closure. Plates were marked on the bottom to ensure that images were captured from the same regions at each time point.

Images of the wound area were obtained at 0 h and 48 h using an inverted microscope (Olympus Corporation, Tokyo, Japan). Wound closure was quantified using ImageJ software by measuring the wound area through threshold-based segmentation. Migration rate was calculated using the following formula:

$$\mathrm{Wound}\;\mathrm{Closure}\;(\%)=(\frac{{\mathrm{Area}}_{0h}-{\mathrm{Area}}_{48h}}{{\mathrm{Area}}_{0h}})\times 100$$

### 2.8. Transwell Migration Assay

Cell migration was assessed using Transwell chambers equipped with 8-µm pore polycarbonate membranes (Corning Inc., Corning, NY, USA). HeLa cells were harvested, resuspended in serum-free DMEM, and 2 × 10^4^ cells were seeded into the upper chamber in a final volume of 200 µL. The lower chamber was filled with 600 µL of DMEM supplemented with 10% FBS, which served as a chemoattractant.

Sitagliptin, doxorubicin, or their combination was added to the upper chamber at the designated concentrations, and the plates were incubated for 48 h at 37 °C in a humidified incubator with 5% CO_2_. After incubation, non-migrated cells remaining on the upper surface of the membrane were gently removed with a cotton swab.

Migrated cells on the lower membrane surface were fixed with methanol for 10 min and stained with 0.1% crystal violet for 20 min. Excess dye was removed by washing with distilled water. Stained cells were counted in five randomly selected microscopic fields under a light microscope.

Migration rate was calculated using the following formula:

$$\mathrm{Migration}\;(\%\;\mathrm{of}\;\mathrm{control})=(\frac{\mathrm{Number}\;\mathrm{of}\;\mathrm{migrated}\;\mathrm{cells}\;(\mathrm{treatment})}{\mathrm{Number}\;\mathrm{of}\;\mathrm{migrated}\;\mathrm{cells}\;(\mathrm{control})})\times 100$$

### 2.9. Transwell Invasion Assay

Cell invasive capacity was evaluated using Matrigel-coated Transwell invasion chambers (Corning BioCoat Matrigel Invasion Chamber; 8-µm pore size). Prior to seeding, inserts were brought to room temperature and the Matrigel layer was hydrated with serum-free medium for 30 min according to the manufacturer’s instructions.

HeLa cells were harvested and resuspended in serum-free DMEM, and 2 × 10^5^ cells were seeded into the upper chamber in a final volume of 200 µL. The lower chamber was filled with 600 µL of DMEM containing 10% FBS, which served as a chemoattractant. Sitagliptin, doxorubicin, or their combination was added to the upper chamber at the designated concentrations. Chambers were incubated for 48 h at 37 °C in a humidified incubator with 5% CO_2_.

Following incubation, non-invaded cells remaining on the upper surface of the membrane were carefully removed with a cotton swab. Cells that invaded through the Matrigel matrix and adhered to the lower membrane surface were fixed with methanol for 10 min and stained with 0.1% crystal violet for 20 min. Excess dye was removed by washing with distilled water.

Invaded cells were counted in five randomly selected microscopic fields, and invasion capacity was calculated as:

$$\mathrm{Invasion}\;(\%\;\mathrm{of}\;\mathrm{control})=(\frac{\mathrm{Invaded}\;\mathrm{cells}\;(\mathrm{treatment})}{\mathrm{Invaded}\;\mathrm{cells}\;(\mathrm{control})})\times 100$$

### 2.10. ELISA Analysis of MMP and TIMP Levels

MMP-1, MMP-2, TIMP-1, and TIMP-2 levels were quantified in culture supernatants using commercial ELISA kits (R&D Systems, Minneapolis, MN, USA) according to the manufacturer’s instructions. HeLa cells were seeded into 6-well plates (3 × 10^5^ cells/well) and treated with sitagliptin, doxorubicin, or their combination for 48 h. At the end of treatment, supernatants were collected and centrifuged at 1500 rpm for 10 min to remove debris, then stored at −80 °C until analysis. Absorbance was measured at 450 nm, and concentrations were calculated from standard curves and expressed in pg/mL.

### 2.11. Normalization of MMP/TIMP Levels

To ensure that reductions in MMP and TIMP levels were not simply due to treatment-induced decreases in cell number, all ELISA results were normalized to the number of viable cells present at the time of supernatant collection. Parallel wells were trypsinized, and viable cell counts were determined using the Trypan blue exclusion method with an automated cell counter (Thermo Fisher, USA). Final concentrations of MMP-1, MMP-2, TIMP-1, and TIMP-2 were expressed as pg per 10^5^ viable cells, ensuring that differences reflected true biological modulation rather than artifacts related to cytotoxicity.

### 2.12. Quantitative Real-Time PCR (qRT-PCR) Analysis of Akt and ERK

HeLa cells were seeded into 6-well plates at a density of 3 × 10^5^ cells per well and treated with sitagliptin, doxorubicin, or their combination for 48 h. Total RNA was extracted using TRIzol reagent (Thermo Fisher Scientific, Waltham, MA, USA) following the manufacturer’s instructions. RNA concentration and purity were assessed using a NanoDrop spectrophotometer (Thermo Fisher Scientific, Waltham, MA, USA), and samples with an A260/A280 ratio between 1.8 and 2.1 were used for downstream analysis. Residual genomic DNA contamination was removed by on-column DNase treatment.

cDNA synthesis was performed from 1 µg of total RNA using the iScript cDNA Synthesis Kit (Bio-Rad Laboratories, Hercules, CA, USA). qRT-PCR was conducted using SYBR Green Master Mix (Applied Biosystems, Foster City, CA, USA) on a Bio-Rad CFX96 real-time PCR detection system. Gene-specific primers for Akt and ERK were designed and validated to ensure amplification efficiency within the range of 95–105%. No-template controls (NTC) and no-reverse-transcriptase controls (no-RT) were included to verify the absence of contamination.

Each 20 µL reaction contained 1 µL cDNA, 10 µL SYBR Green Master Mix, and 0.5 µM forward and reverse primers. The thermal cycling conditions were as follows:

1. Initial denaturation at 95 °C for 3 min;
2. 40 cycles of:

−95 °C for 10 s;

−60 °C for 30 s.

A melting curve analysis was performed to confirm the specificity of each amplification product. Relative gene expression levels were calculated using the 2^−ΔΔCt^ method, with GAPDH used as the endogenous reference gene. All samples were run in technical triplicates, and data were expressed as fold changes relative to the control group.

The gene-specific primer sequences used for the amplification of Akt, ERK1/2, and GAPDH transcripts are listed in Table 1, along with their corresponding forward and reverse primer orientations. All primers were designed to span exon–exon junctions to prevent amplification of genomic DNA, and each primer pair demonstrated an amplification efficiency between 95% and 105%.

### 2.13. Bioinformatics Analyses

Bioinformatic analyses were performed to identify potential molecular targets and shared signaling pathways associated with sitagliptin and doxorubicin. Putative target proteins were obtained from PubChem, SwissTargetPrediction (v2024), and DrugBank (v5.1.10) databases using default prediction parameters. After redundant entries were removed, the overlapping targets of both drugs were identified.

The predicted and overlapping molecular targets of sitagliptin and doxorubicin are summarized in Table 2.

Protein–protein interaction (PPI) networks were constructed using STRING (version 12.0) with a medium confidence score ≥ 0.4 and limited to Homo sapiens. Additional interaction data and functional associations were validated using GeneMANIA. Network topological features (degree, betweenness, and clustering coefficient) were analyzed, and interaction maps were visualized using Cytoscape 3.9.1.

Gene Ontology (GO) biological processes and KEGG pathway enrichment analyses were performed using DAVID (v2024). An enrichment threshold of *p* < 0.05, corrected using Benjamini–Hochberg false discovery rate (FDR), was applied. Enrichment outputs highlighted significant involvement of the MAPK, PI3K/Akt, ECM remodeling (MMP/TIMP), oxidative stress, and apoptosis pathways. These bioinformatic findings were integrated with experimental data to elucidate the potential mechanisms underlying the synergistic effects of the sitagliptin–doxorubicin combination.

### 2.14. Statistical Analysis

All experiments were conducted with at least three independent biological replicates, and each measurement within an assay was performed in technical triplicates. Data are presented as the mean ± standard error of the mean (SEM). The normality of data distribution was assessed using the Kolmogorov–Smirnov test, and homogeneity of variances was evaluated using Levene’s test.

For comparisons among multiple groups, one-way ANOVA followed by Tukey’s post hoc test was applied. A *p*-value of <0.05 was considered statistically significant. IC_50_ values and nonlinear dose–response curves were generated using a four-parameter logistic regression model in GraphPad Prism 9.0.

CI values and isobologram analyses were obtained automatically using CompuSyn software (ComboSyn Inc., USA) based on the Chou–Talalay method. All statistical analyses (except CI calculations) were performed using SPSS version 28.0 (IBM Corp., Armonk, NY, USA).

## 3. Results

### 3.1. Effects of Sitagliptin and Doxorubicin on Cell Viability

**Figure 1:**
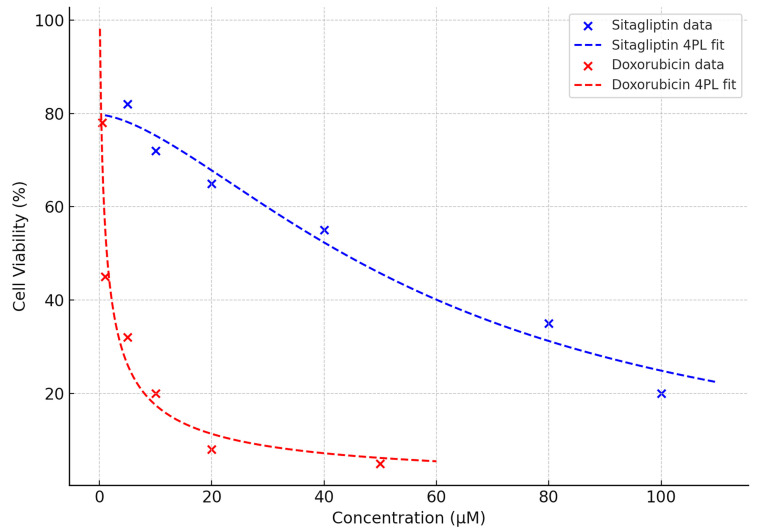
Dose–response curves of sitagliptin and doxorubicin in HeLa cells with overlaid four-parameter logistic (4PL) regression fits. HeLa cells were treated for 48 h with increasing concentrations of sitagliptin (5–100 µM) or doxorubicin (0.5–50 µM), and cell viability was assessed by the MTT assay. Experimental data points (mean values) are shown for each drug. The dashed curves represent the fitted 4PL nonlinear regression models used to calculate IC_50_ values. The fitted curves demonstrate strong agreement with the experimental data and validate the appropriateness of the 4PL model for estimating IC_50_. Sitagliptin exhibited an IC_50_ of 52.4 µM, while doxorubicin showed an IC_50_ of 4.18 µM, confirming the differential potency of the two agents in HeLa cells.

HeLa cells were treated with sitagliptin, doxorubicin, or their combination for 48 h, and cell viability was assessed using the MTT assay (Figure 1). Both agents significantly reduced cell viability in a dose-dependent manner. Sitagliptin exhibited mild cytotoxicity at lower concentrations (5–20 µM), whereas a marked reduction in viability was observed at concentrations ≥ 40 µM. In contrast, doxorubicin displayed potent cytotoxicity even at low doses, with viability significantly declining from 1 µM onward and falling below 50% at concentrations higher than 10 µM (*p* < 0.001). These findings confirm the differential sensitivity of HeLa cells to sitagliptin and doxorubicin and support subsequent analyses of their combined therapeutic potential.

### 3.2. Synergism Findings

**Figure 2:**
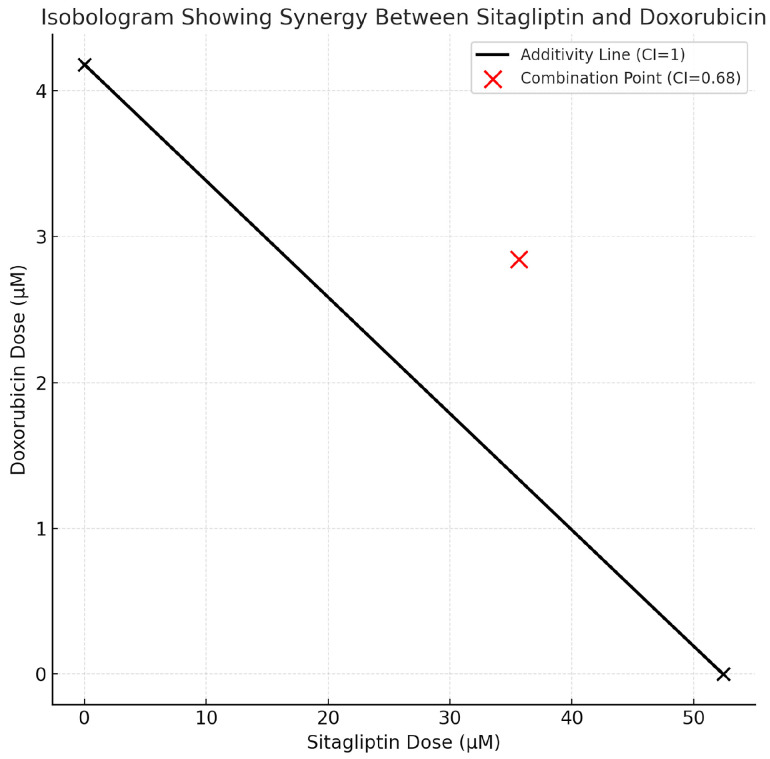
Isobologram illustrating the interaction between sitagliptin and doxorubicin based on the Chou–Talalay method. The black line represents the theoretical line of additivity (CI = 1), connecting the individual IC_50_ values of sitagliptin (52.4 µM) and doxorubicin (4.18 µM). The red combination point corresponds to the experimentally observed dose pair at the FA_50_ effect level (CI = 0.68), which falls below the additivity line, confirming a synergistic interaction between the two agents. According to the Chou–Talalay model, CI < 1 defines synergy, and the CI value represents a calculated index at a given effect level rather than a separate “synergistic line”. This graphical representation supports the CI analysis and provides visual validation of the synergistic effect observed in the combination treatment.

The combined effect of sitagliptin and doxorubicin was evaluated using the Chou–Talalay method. Dose–response data entered into CompuSyn software generated a CI of 0.68 at the IC_50_ effect level, indicating a clear synergistic interaction between the two agents (Figure 2). Isobologram plots further supported this synergy, as all combination data points fell below the line of additivity, demonstrating that the combined treatment achieved greater cytotoxicity than either drug alone at equivalent fractional effect levels.

At IC_50_-based fixed-ratio dosing, the sitagliptin–doxorubicin combination reduced HeLa cell viability by approximately 70%, a significantly greater reduction than observed with single-agent treatments (*p* < 0.001). These results confirm that sitagliptin enhances the anticancer activity of doxorubicin, supporting the hypothesis that this combination reduces cell viability more effectively and may potentiate downstream mechanisms such as ROS-mediated apoptosis.

**Figure 3:**
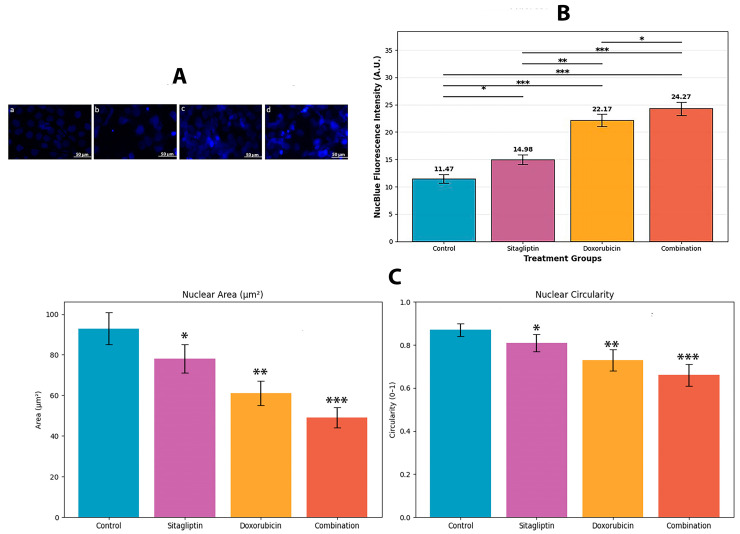
Nuclear morphological alterations and quantitative analysis following 48 h treatments with sitagliptin, doxorubicin, or their combination. (**A**) Representative NucBlue-stained fluorescence images showing chromatin condensation and nuclear fragmentation across treatment groups. (**B**) Quantification of NucBlue fluorescence intensity (A.U.), demonstrating significantly increased nuclear condensation in all treatment groups, with the greatest effect observed in the combination group. (**C**) Nuclear area and nuclear circularity measurements obtained by ImageJ analysis. Both parameters decreased progressively from single-agent treatments to combination therapy, indicating enhanced nuclear fragmentation and apoptotic morphology. Data represent mean ± SEM (n = 3). Statistical significance: * *p* < 0.05, ** *p* < 0.01, *** *p* < 0.001.

Significant apoptosis-associated nuclear changes were observed during the 48 h incubation. As shown in Figure 3A, NucBlue staining revealed marked chromatin condensation and nuclear fragmentation in all treatment groups, with the most pronounced alterations occurring in the combination-treated cells. These qualitative nuclear morphological changes indicate that the sitagliptin–doxorubicin combination elicits a stronger apoptotic response than either agent alone.

Quantitative analysis of NucBlue fluorescence intensity (Figure 3B) further supported these observations. The combination therapy produced the highest fluorescence signal (24.27 ± 0.58 AU), which was significantly greater than that induced by sitagliptin (14.98 ± 0.51 AU, *p* < 0.001) or doxorubicin alone (22.17 ± 0.63 AU, *p* < 0.05).

To complement these findings, nuclear area and circularity were quantified using ImageJ (Figure 3C). Both parameters decreased progressively from single-agent treatments to the combination group (Control: 93 µm^2^ and 0.87; Sitagliptin: 78 µm^2^ and 0.81; Doxorubicin: 61 µm^2^ and 0.73; Combination: 49 µm^2^ and 0.66), consistent with enhanced chromatin condensation and apoptotic nuclear fragmentation. These reductions were statistically significant compared with control (*p* < 0.05 to *p* < 0.001).

Collectively, these qualitative and quantitative metrics demonstrate that the combined treatment induces a more robust apoptotic response than either single agent, as evidenced by intensified nuclear condensation, reduced nuclear size, and decreased circularity.

### 3.3. Effects on ROS Production

**Figure 4:**
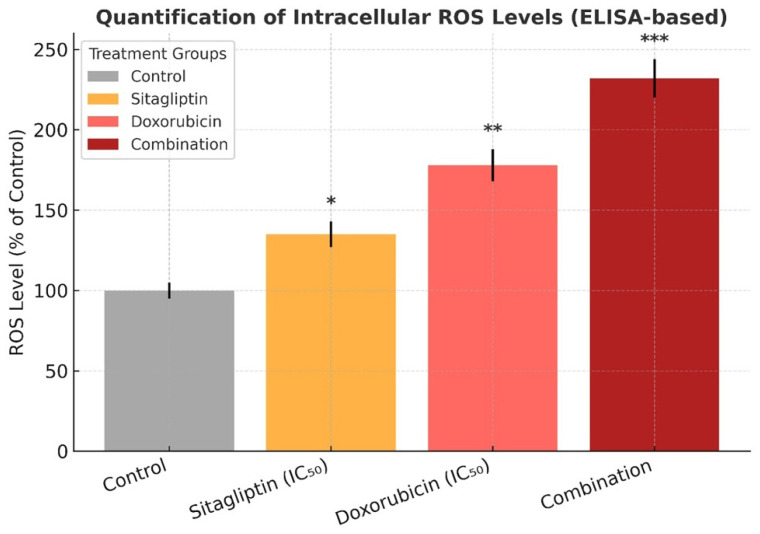
Quantification of intracellular ROS levels in HeLa cells after sitagliptin and doxorubicin treatments. HeLa cells were treated with sitagliptin (IC_50_ = 52.4 µM), doxorubicin (IC_50_ = 4.18 µM), or their combination for 48 h, and intracellular ROS levels were measured using a DCFDA-based fluorometric assay. Fluorescence values were normalized to the control group and expressed as percentages. Data are shown as mean ± SEM (n = 3). Statistical significance was assessed using one-way ANOVA with Tukey’s post hoc test (* *p* < 0.05, ** *p* < 0.01, *** *p* < 0.001 vs. control).

Intracellular ROS levels were assessed using the DCFDA fluorescence assay. Treatment with sitagliptin or doxorubicin alone induced a mild to moderate increase in ROS generation compared with the control group (*p* < 0.05). In contrast, the combination treatment produced a marked elevation in ROS levels, resulting in nearly a twofold increase in fluorescence intensity relative to either monotherapy (*p* < 0.001, Figure 4). This substantial rise in oxidative stress indicates that the combined treatment amplifies ROS-mediated cellular damage and likely contributes to the enhanced apoptotic response observed in subsequent analyses.

### 3.4. Apoptosis Analysis Results (Annexin V/PI, Caspase Activity)

**Figure 5:**
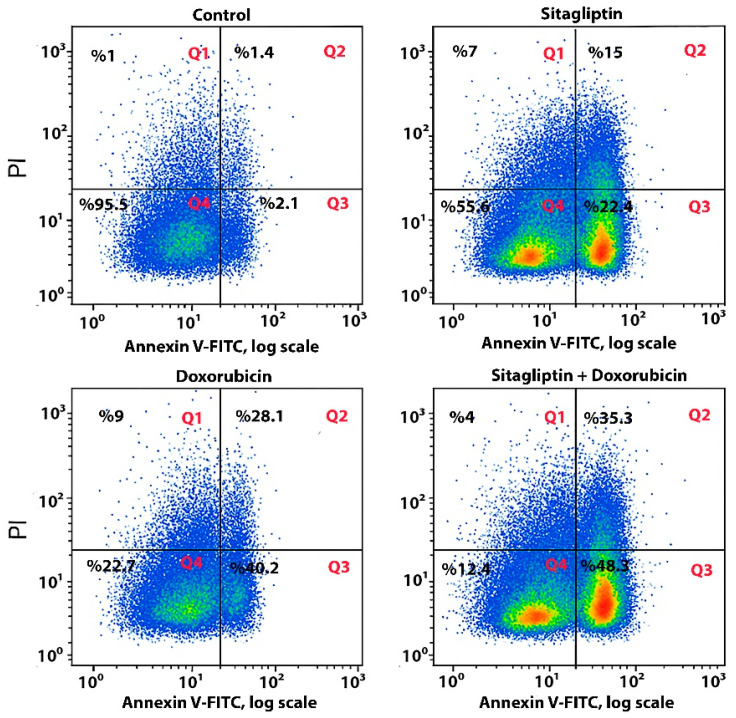
Representative dot plots of Annexin V–FITC (x-axis, log scale) and PI (y-axis, log scale) staining in HeLa cells following 48 h treatment with control, sitagliptin (IC_50_), doxorubicin (IC_50_), or their combination. Quadrants indicate distinct cell populations: Q1 (Annexin V^−^/PI^+^), necrotic; Q2 (Annexin V^+^/PI^+^), late apoptotic; Q3 (Annexin V^+^/PI^−^), early apoptotic; and Q4 (Annexin V^−^/PI^−^), viable cells. For the control group, the distribution was Q1: 1.0%, Q2: 1.4%, Q3: 2.1%, Q4: 95.5%, reflecting predominantly viable cells. Under combination treatment, apoptotic fractions increased markedly (Q2 + Q3), consistent with enhanced apoptosis. Plots represent one of three independent experiments with similar results. Quadrant gates were defined using untreated control cells and applied consistently to all samples.

Flow cytometric analysis using Annexin V/PI staining demonstrated a pronounced increase in apoptosis following combination treatment. The sitagliptin + doxorubicin group exhibited a total apoptotic rate of approximately 45%, which was significantly higher than the levels observed in the single-agent groups (15–25%, *p* < 0.001; Figure 5). Both early and late apoptotic populations contributed to this elevation, indicating that the combination therapy triggers apoptosis more effectively than either drug alone.

**Figure 6:**
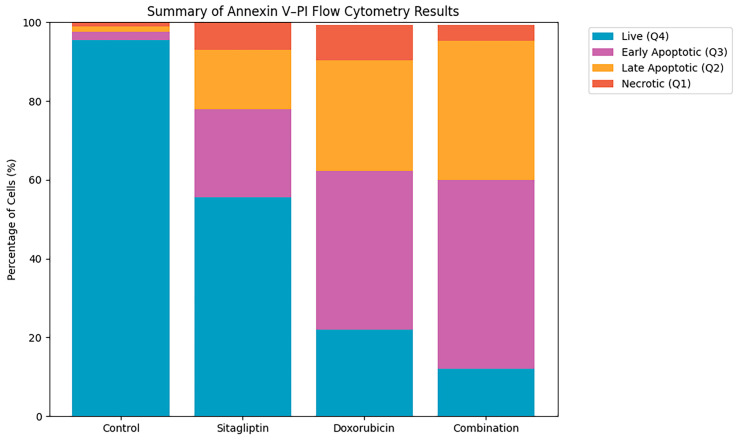
Summary of Annexin V–PI flow cytometry analysis. Stacked bar graph showing the distribution of live (Q4), early apoptotic (Q3), late apoptotic (Q2), and necrotic (Q1) cell populations across treatment groups. Combination therapy resulted in a substantial shift from live to apoptotic populations, consistent with enhanced apoptosis induction.

Flow cytometry analysis demonstrated a marked redistribution of cell populations following treatment (Figure 6). In the control group, the majority of cells remained viable (95.5%). Sitagliptin and doxorubicin each reduced the proportion of live cells to 55.6% and 22.0%, respectively, accompanied by corresponding increases in early and late apoptosis. Combination therapy produced the most pronounced effect, reducing viable cells to 12.0% and yielding the highest levels of both early (48.0%) and late (35.3%) apoptotic populations. These results confirm that the sitagliptin–doxorubicin combination induces significantly stronger apoptotic responses than either agent alone.

**Figure 7:**
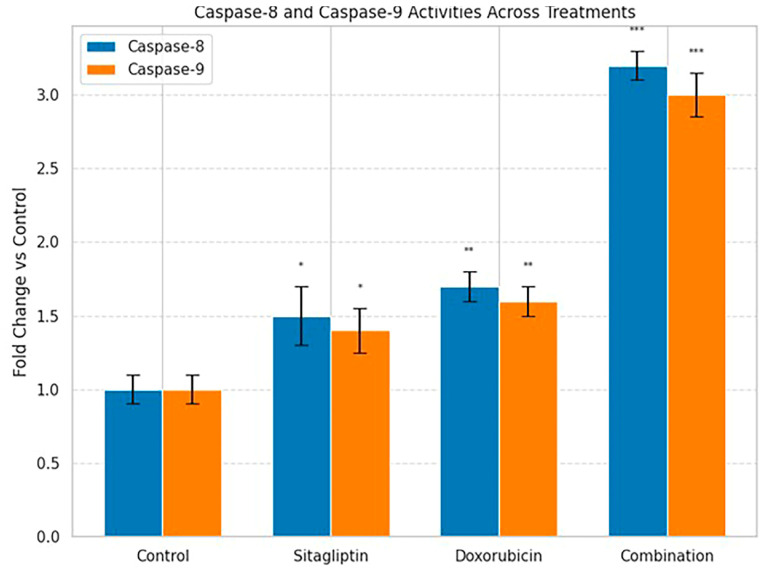
Caspase-8 and caspase-9 activity levels following sitagliptin and doxorubicin treatments. Fold changes in caspase-8 and caspase-9 activities were quantified relative to untreated control cells after 48 h treatment with sitagliptin, doxorubicin, or their combination. Data are presented as mean ± SEM (n = 3). Both enzymes showed significant activity increases, with the combination treatment inducing the highest activation, consistent with enhanced apoptotic signaling. Statistical significance vs. control: * *p* < 0.05, ** *p* < 0.01, *** *p* < 0.001.

In the caspase activity analysis, the combination treatment led to a marked elevation in both caspase-8 and caspase-9 activities, indicating robust activation of the extrinsic and intrinsic apoptotic pathways (Figure 7). The significant increase in these initiator caspases aligns with the heightened ROS generation observed earlier and supports the conclusion that oxidative stress amplifies apoptotic signaling under combination therapy.

### 3.5. Transwell Migration and Invasion Findings

#### 3.5.1. Migration Assay

**Figure 8:**
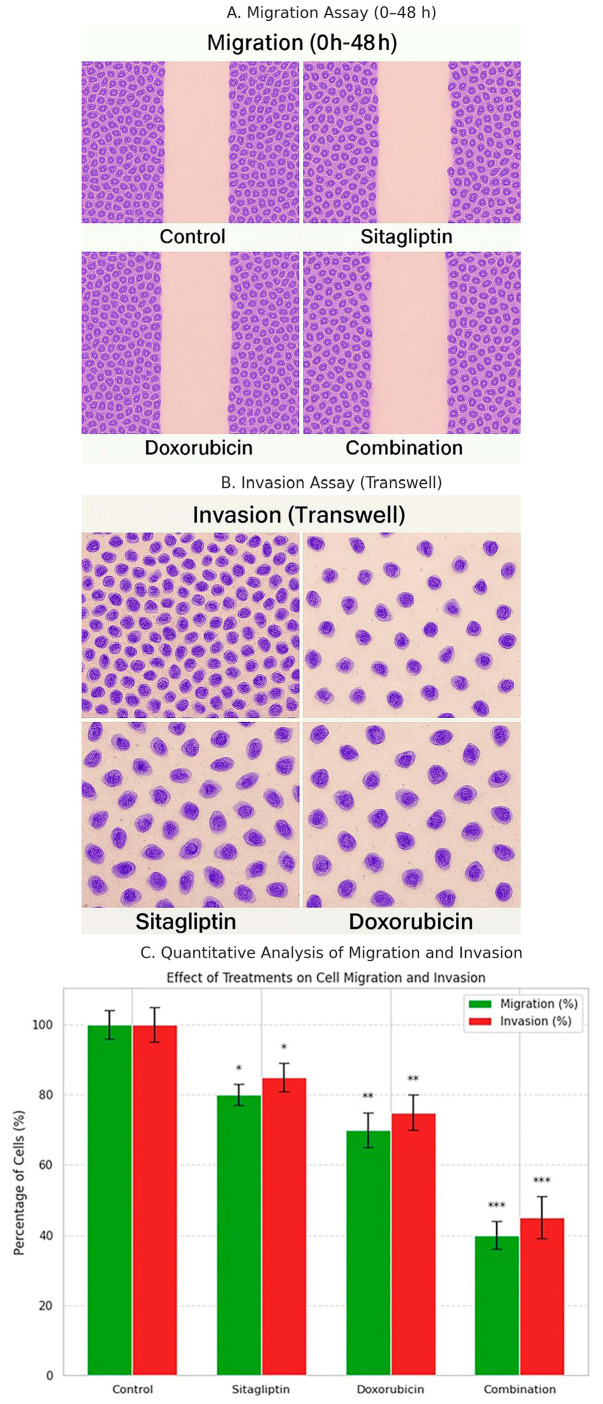
Effects of sitagliptin, doxorubicin, and their combination on HeLa cell migration and invasion. (**A**) Representative brightfield images from the wound healing migration assay at 0 h and 48 h for each treatment group. The control group demonstrated nearly complete wound closure, whereas sitagliptin and doxorubicin treatments reduced migration capacity. The combination treatment resulted in the least wound closure, indicating a strong inhibitory effect on cell motility. (**B**) Representative images of invaded cells on crystal violet–stained Transwell membranes. The number of invading cells decreased in all treatment groups compared with the control, with the combination treatment showing the most pronounced reduction. (**C**) Quantitative analysis of migration and invasion percentages. Both assays showed significant decreases in migration and invasion following sitagliptin or doxorubicin monotherapy, with the greatest suppression observed under combination treatment. Data are presented as mean ± SEM. Statistical significance: * *p* < 0.05, ** *p* < 0.01, *** *p* < 0.001.

Representative images from the wound healing migration assay are shown in Figure 8A. Both sitagliptin and doxorubicin monotherapies modestly reduced the migratory capacity of HeLa cells, resulting in an approximately 20–30% decrease in wound closure compared with the control group. In contrast, the combination treatment produced a markedly stronger inhibitory effect, reducing cell migration by approximately 60% (*p* < 0.001), as quantified in Figure 8C. These findings indicate that the combined therapy impairs cell motility more effectively than either agent alone. 

#### 3.5.2. Invasion Assay

Representative images from the Matrigel-coated Transwell invasion assay are presented in Figure 8B. Consistent with the migration results, the combination treatment caused a pronounced reduction in invasive capacity. Quantitative analysis demonstrated a 55–60% decrease in invasion compared with the control group, which was significantly greater than the effects observed with single-agent treatments (*p* < 0.001; Figure 8C). These results indicate that sitagliptin enhances the anti-invasive activity of doxorubicin, leading to a substantial suppression of metastatic potential.

### 3.6. MMP/TIMP ELISA Findings

**Figure 9:**
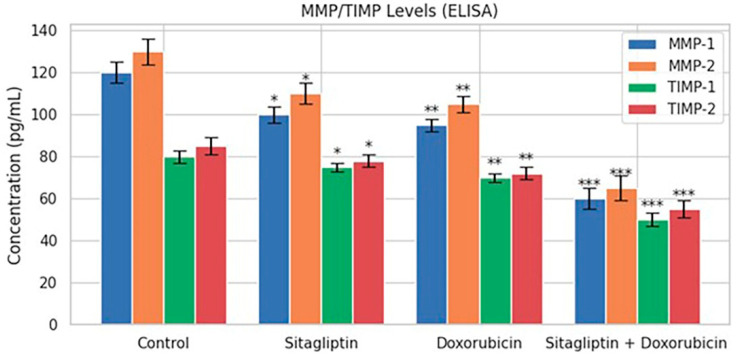
MMP-1, MMP-2, TIMP-1, and TIMP-2 levels measured by ELISA after 48 h treatments. Data are presented as mean ± SEM (n = 3). Statistical significance relative to the control group is indicated as * *p* < 0.05, ** *p* < 0.01, *** *p* < 0.001. In addition, direct comparisons between doxorubicin and combination treatments revealed significant reductions in the combination group for all four markers: MMP-1 (** *p* < 0.01), MMP-2 (*** *p* < 0.001), TIMP-1 (** *p* < 0.01), and TIMP-2 (** *p* < 0.01). These results support the synergistic inhibitory effect of the combined therapy.

ELISA analyses demonstrated that the combined sitagliptin–doxorubicin treatment resulted in a significant reduction in the secretion of MMP-1, MMP-2, TIMP-1, and TIMP-2 compared with the control group (*p* < 0.01; Figure 9). In contrast, single-agent treatments produced only modest decreases in these markers, indicating that the combination exerts a stronger inhibitory effect on extracellular matrix remodeling processes associated with metastasis and cell migration.

Importantly, because all ELISA values were normalized to viable cell counts, the observed decreases in MMP and TIMP levels were confirmed to be independent of cytotoxicity-related changes in cell number. Normalization preserved the overall suppression pattern, verifying that the reductions reflected genuine biological modulation rather than artifacts arising from decreased cell density.

### 3.7. Akt/ERK mRNA Expression (qRT-PCR)

**Figure 10:**
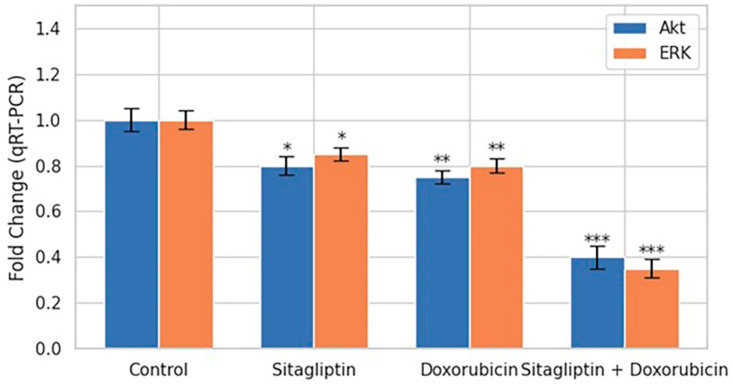
Effects of sitagliptin, doxorubicin, and their combination on Akt and ERK mRNA expression. Relative mRNA levels of Akt and ERK were quantified by qRT-PCR after 48 h treatments and expressed as fold change versus control. Data represent mean ± SEM (n = 3). Both agents individually reduced Akt and ERK expression, while the combination treatment produced the strongest downregulation, indicating suppression of PI3K/Akt and MAPK/ERK signaling. Statistical significance vs. control: * *p* < 0.05, ** *p* < 0.01, *** *p* < 0.001.

qRT-PCR analysis revealed that the combination of sitagliptin and doxorubicin produced a marked suppression of Akt and ERK mRNA expression compared with the control group (Figure 10). In contrast, sitagliptin or doxorubicin alone induced only modest decreases in the transcript levels of these signaling molecules. The pronounced downregulation observed with the combination therapy indicates a more effective inhibition of the PI3K/Akt and MAPK/ERK pathways, supporting suppression of key survival-associated signaling pathways in HeLa cells under dual treatment.

### 3.8. Bioinformatic Findings

Target prediction analyses performed through PubChem, SwissTargetPrediction, and DrugBank identified multiple overlapping protein targets for sitagliptin and doxorubicin. Protein–protein interaction (PPI) mapping using STRING and GeneMANIA revealed that both agents converge on key regulatory nodes within the MMP, caspase (CASP), and MAPK signaling pathways, indicating shared mechanistic activity.

**Figure 11:**
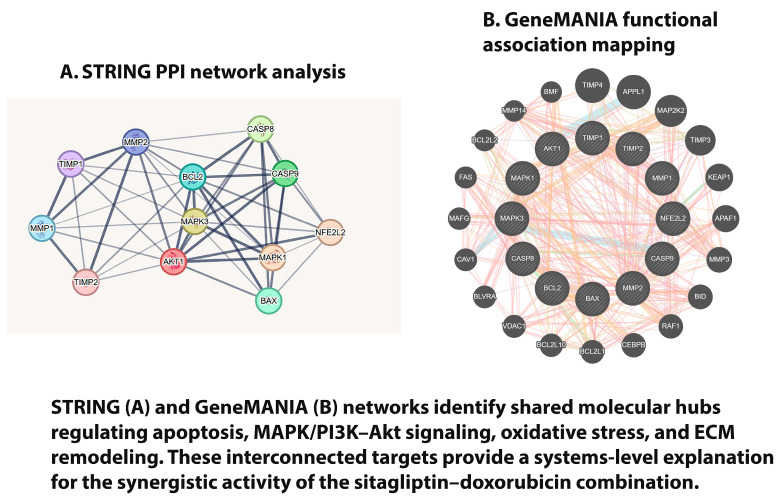
STRING (**A**) and GeneMANIA (**B**) network analyses reveal shared molecular hubs regulating apoptosis, MAPK/PI3K–Akt signaling, oxidative stress, and ECM remodeling, supporting the mechanistic basis of the sitagliptin–doxorubicin synergy.

STRING PPI network analysis (Figure 11A) highlighted a densely interconnected structure with several high-degree central nodes, including AKT1, MAPK1/3, CASP8, CASP9, BAX, and MMP/TIMP family members, reflecting their coordinated involvement in apoptosis, ECM remodeling, oxidative stress responses, and cell survival pathways.

GeneMANIA functional association mapping (Figure 11B) confirmed these interactions and identified additional co-expression and pathway-level associations linking oxidative stress regulation (NFE2L2), apoptotic activation, and metastatic behavior.

Cytoscape visualization of the integrated network demonstrated tight clustering among nodes regulating apoptotic signaling, redox balance, and metastatic potential, further supporting the interconnected nature of these pathways.

**Figure 12:**
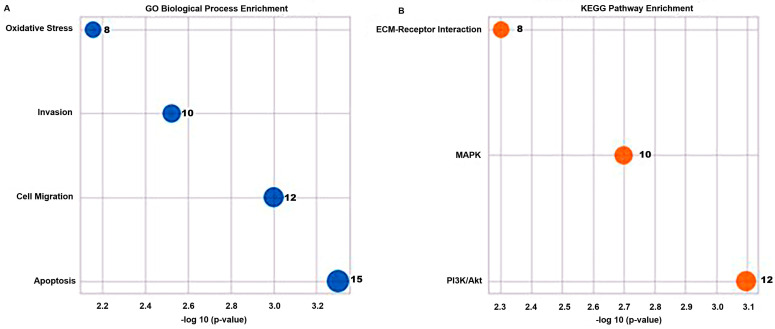
Bioinformatic enrichment analysis of Sitagliptin and Doxorubicin targets. (**A**) GO Biological Process enrichment of shared Sitagliptin–Doxorubicin target genes, demonstrating significant enrichment in apoptosis, cell migration, invasion, and oxidative stress processes. Dot size represents the number of genes associated with each term, and the x-axis indicates −log10(*p*-value). (**B**) KEGG pathway enrichment analysis showing PI3K/Akt, MAPK, and ECM–receptor interaction as major pathways regulated by both drugs. Dot size corresponds to gene count within each pathway. These analyses support that Sitagliptin and Doxorubicin synergistically modulate key signaling networks involved in proliferation, survival, and metastasis. In both panels (**A**,**B**), the x-axis represents the −log10-transformed p-values, where higher values indicate greater statistical significance.

GO (Gene Ontology) enrichment analysis demonstrated that common targets were predominantly associated with biological processes involved in apoptosis, cell migration, invasion, and oxidative stress (Figure 12A). KEGG pathway analysis further highlighted the involvement of PI3K/Akt, MAPK, and ECM–receptor interaction pathways, suggesting that the combination therapy may modulate tumor progression and metastatic behavior by simultaneously affecting these interconnected networks (Figure 12B).

Collectively, these bioinformatic results closely mirror the experimental observations and support a systems-level mechanistic model in which the sitagliptin–doxorubicin combination enhances ROS generation, disrupts MMP/TIMP homeostasis, activates caspase-dependent apoptosis, and suppresses PI3K/Akt–MAPK signaling. These converging effects provide a comprehensive molecular explanation for the synergistic anticancer activity observed in vitro.

## 4. Discussion

Our study demonstrates that the combination of sitagliptin and doxorubicin exerts a synergistic anticancer effect in HeLa cervical cancer cells by simultaneously modulating apoptosis, oxidative stress, metastasis-associated proteases, and key intracellular signaling pathways. Annexin V/PI flow cytometry revealed that the combined treatment significantly increased apoptotic cell death compared with either agent alone, indicating a potentiation of programmed cell death mechanisms (Figure 4). This enhanced apoptotic response was further supported by the marked activation of caspase-8 and caspase-9, suggesting that both the extrinsic and intrinsic pathways are involved in mediating the observed synergy [16,17,18,19].

Parallel assessments of oxidative stress demonstrated a pronounced increase in intracellular ROS levels following combined treatment. This ROS elevation is likely a key driver of mitochondrial dysfunction and subsequent caspase activation, consistent with previous reports that identify oxidative stress as a central trigger of both caspase-dependent and caspase-independent apoptosis [20,21]. Notably, the DCFDA assay showed that the combination regimen induced an approximately two-fold increase in ROS levels relative to the untreated control, while producing a statistically significant but more moderate increase compared with doxorubicin alone (Figure 4). This surge in ROS appears to serve as more than a secondary cellular response; instead, it likely represents a primary mechanistic determinant of the synergistic cytotoxicity. It is important to note that the effects of sitagliptin on intracellular reactive oxygen species are not uniform across experimental models. Several studies, particularly those conducted in non-malignant tissues or in cardiometabolic injury models, have reported that sitagliptin reduces oxidative stress by enhancing antioxidant defenses and suppressing ROS generation [22,23]. In contrast, emerging evidence from cancer cell models suggests that, at higher micromolar concentrations and under conditions of oncogenic stress, sitagliptin may disrupt redox homeostasis and promote ROS accumulation, thereby sensitizing tumor cells to apoptosis. These context-dependent differences likely reflect variations in cell type, basal redox state, DPP-4 expression, and experimental dosing, underscoring that sitagliptin can exert either antioxidant or pro-oxidant effects depending on the biological setting.

The associated nuclear damage observed in the NucBlue staining analysis reinforces this interpretation. The combination group exhibited strikingly increased fluorescence intensity (24.27 AU) relative to the control (11.47 AU) and single-agent treatments, indicating extensive chromatin condensation and nuclear fragmentation, hallmark features of late-stage apoptosis [24,25]. These morphological alterations correlate strongly with the increased percentage of Annexin V+/PI+ cells detected by flow cytometry. Collectively, these results suggest a mechanistic sequence in which sitagliptin enhances cellular susceptibility to doxorubicin-induced ROS, pushing cells beyond their oxidative stress tolerance threshold. This “redox priming” effect may arise from sitagliptin-induced disruption of cellular redox homeostasis, thereby amplifying doxorubicin’s cytotoxic potency [22,23]. Because the MTT assay reflects mitochondrial dehydrogenase activity, doxorubicin-induced mitochondrial suppression may partially contribute to reduced MTT signal in viable but metabolically impaired cells. To avoid overinterpreting this reduction as cell death alone, MTT findings were interpreted alongside Annexin V/PI cytometry results, which showed a highly consistent apoptotic pattern across treatment groups. Future studies assessing mitochondrial membrane potential and key redox regulators (e.g., Nrf2, GST) could further clarify the molecular intermediates involved in this process.

In addition to its pro-apoptotic effects, the combination treatment also demonstrated robust anti-metastatic activity. Both migration and invasion assays showed that sitagliptin and doxorubicin together markedly suppressed cell motility and invasiveness compared to monotherapy. This inhibition correlated with significant decreases in MMP-1, MMP-2, TIMP-1, and TIMP-2 levels, as confirmed by ELISA analysis (Figure 9). Importantly, these ELISA results were normalized to viable cell counts to exclude any bias due to treatment-induced cytotoxicity. The suppression of MMP/TIMP expression aligns with prior studies demonstrating that modulation of extracellular matrix remodeling enzymes can effectively impair metastatic progression in various cancer models [26,27].

Because treatment-induced apoptosis has the potential to confound migration and invasion assays, we considered whether reduced cell viability might partially account for the observed suppression of motility. However, the migration and invasion experiments were performed over a shorter 24 h period, during which Annexin V/PI analysis showed minimal apoptosis, whereas substantial apoptotic activity emerged only after 48 h. This temporal separation indicates that the decrease in motility is not a secondary consequence of cell death. Furthermore, our findings align closely with those of Beckenkamp et al. [9], who demonstrated that sitagliptin reduces migration and adhesion in cervical cancer cells independently of cytotoxicity or DPPIV/CD26 expression. Taken together, these results support that the anti-migratory and anti-invasive effects observed in our study reflect true modulation of metastatic pathways rather than viability-related artifacts. These observations are further supported by a recent systematic review integrating in vitro, in vivo, and in silico evidence on the role of DPP-4 and its inhibitors in female malignancies [8]. This review highlighted that cervical cancer appears particularly sensitive to alterations in DPP-4 expression, with DPP-4 overexpression associated with enhanced migration and proliferation, whereas pharmacological inhibition using sitagliptin was reported to reduce cell adhesion in cervical cancer models. Importantly, the authors emphasized that the effects of DPP-4 inhibitors are highly cancer-type–specific and may involve both DPP-4–dependent and independent mechanisms. In this context, our findings extend the existing literature by demonstrating that sitagliptin, especially when combined with doxorubicin, suppresses migration and invasion in HeLa cells while concomitantly modulating MMP/TIMP balance and oxidative stress–associated apoptotic signaling.

Interestingly, the decrease in TIMP levels warrants careful interpretation. Although TIMPs classically function as endogenous inhibitors of MMPs, they also exhibit context-dependent roles in regulating cell proliferation, apoptosis, and tissue remodeling [15,28]. Consequently, the biological significance of reduced TIMP expression under combination therapy requires further investigation using functional assays such as MMP-specific inhibition or TIMP-mutant constructs to delineate precise downstream outcomes.

Our findings also highlight the impact of combination therapy on intracellular signaling pathways essential for cancer cell survival and metastasis. The combination treatment significantly downregulated the mRNA expression of Akt and ERK (Figure 10), two central components of the PI3K/Akt and MAPK/ERK pathways. These pathways regulate diverse cellular processes including proliferation, growth, resistance to apoptosis, and metastatic potential [29,30]. Although the transcriptional suppression detected by qRT-PCR provides important insights, validation at the protein and phosphorylation level remains crucial. Bioinformatic analyses using STRING and GeneMANIA supported these experimental findings by showing that sitagliptin and doxorubicin converge on overlapping regulatory nodes involved in apoptosis, migration, and oxidative stress responses [31]. Together, these data suggest that the synergistic anticancer effects arise from multi-level interference across redox signaling, protease regulation, and canonical survival pathways.

Taken together, our findings suggest a multifaceted mechanism whereby sitagliptin enhances the cytotoxic and antimetastatic effects of doxorubicin through (i) potent induction of ROS-mediated apoptosis, (ii) suppression of MMP/TIMP-associated invasive behavior, and (iii) coordinated inhibition of the PI3K/Akt and MAPK/ERK pathways. Such multimodal interference may help overcome chemoresistance and reduce metastatic potential, consistent with prior combinatorial therapeutic strategies targeting overlapping proliferative and invasive mechanisms [32,33]. Nonetheless, the role of DPP-4 inhibitors in cancer biology appears to be context-dependent, with some studies suggesting protumorigenic effects due to immunomodulation or enhanced cell survival [4,7,34,35]. These conflicting findings highlight the need for a nuanced assessment of sitagliptin’s therapeutic relevance across different cancer types and microenvironmental contexts [36].

Consistent with this context-dependent framework, when compared with findings from other cancer types, the effects of sitagliptin appear to be highly dependent on cellular context and experimental dosing. In breast and lung cancer models, DPP-4 inhibition has been reported to exert both tumor-suppressive and tumor-promoting effects, including modulation of apoptosis, migration, and immune-related pathways, depending on DPP-4 expression levels and microenvironmental factors [4,7,8,36]. Importantly, several in vitro anticancer studies investigating sitagliptin and other DPP-4 inhibitors have employed micromolar concentrations that exceed those achieved under standard antidiabetic dosing, similar to the concentrations used in the present study. These higher concentrations are commonly required to elicit direct cytotoxic, pro-oxidative, or anti-migratory effects in cancer cells, whereas antidiabetic plasma levels primarily reflect systemic metabolic regulation rather than direct tumor targeting. In this context, our sitagliptin concentrations are comparable to those reported in other in vitro anticancer studies and should be interpreted as mechanistic rather than pharmacokinetic equivalents of clinical dosing.

While our in vitro findings provide strong evidence for the synergistic anticancer effects of sitagliptin and doxorubicin in cervical cancer cells, several limitations must be acknowledged. First, all experiments were performed on a single HeLa cell line. Although HeLa cells are widely used and well-characterized, incorporating additional cervical cancer cell lines with distinct genetic backgrounds (e.g., SiHa, CaSki) would strengthen the generalizability of the findings. Second, the translational relevance is limited by the absence of in vivo validation. Future studies employing xenograft or orthotopic animal models will be necessary to assess the in vivo efficacy, toxicity, and potential pharmacokinetic interactions between sitagliptin and doxorubicin.

Another important limitation relates to drug concentrations. The observed synergistic effects occurred at relatively high sitagliptin concentrations (IC_50_ = 52.4 µM), whereas typical therapeutic plasma levels in humans are considerably lower (approximately 4–8 µM). Although tissue-specific accumulation or alternative dosing strategies such as metronomic or high-dose pulse regimens may theoretically achieve higher local concentrations, this possibility remains hypothetical. Additionally, potential pharmacokinetic or pharmacodynamic interactions between sitagliptin and doxorubicin were not explored in this study. Because sitagliptin has demonstrated both pro- and anti-tumor properties depending on cellular context, a more comprehensive assessment of its immunomodulatory effects is warranted before clinical translation.

Furthermore, although the combination treatment significantly reduced AKT and ERK mRNA levels, the lack of Western blot validation to determine whether corresponding changes occur at the protein or phospho-protein level represents an additional limitation. Protein-level assays could not be performed due to practical constraints, including limited funding; therefore, our conclusions regarding AKT and ERK suppression are restricted to transcriptional regulation. Nevertheless, the close agreement between the mRNA findings and the functional outcomes observed in apoptosis, migration, invasion, and MMP/TIMP expression supports the biological relevance of this transcriptional effect. Future studies incorporating total and phosphorylated protein analyses will be essential to more fully elucidate the mechanistic contribution of AKT/ERK signaling to the synergistic effects of the combination treatment. Additionally, although the observed cellular responses suggest that oxidative stress may contribute to the synergistic effects of sitagliptin and doxorubicin, this mechanism was not directly validated. Experiments with reactive oxygen species–scavenging agents such as N-acetylcysteine (NAC) could not be performed due to resource limitations; therefore, any proposed involvement of oxidative stress remains speculative. Future studies incorporating NAC or other redox modulators will be necessary to determine whether the synergistic response is mediated through ROS-dependent pathways. Finally, the study relied on a single 48 h MTT time point, which is widely used for IC_50_ determination but does not capture temporal variations in drug response. Future studies incorporating multiple time-point viability assays will help clarify the kinetics of the synergistic interaction.

## 5. Conclusions

In conclusion, our findings provide strong in vitro evidence that sitagliptin significantly enhances the anticancer efficacy of doxorubicin through multiple, converging mechanistic pathways. The combination treatment produced a clear synergistic effect (CI < 1), characterized by amplified ROS generation, activation of caspase-8/9–mediated apoptotic signaling, and pronounced downregulation of the Akt/ERK axis. These molecular alterations were accompanied by marked reductions in MMP-1/2 and TIMP-1/2 expression, ultimately suppressing cell migration and invasion.

**Figure 13:**
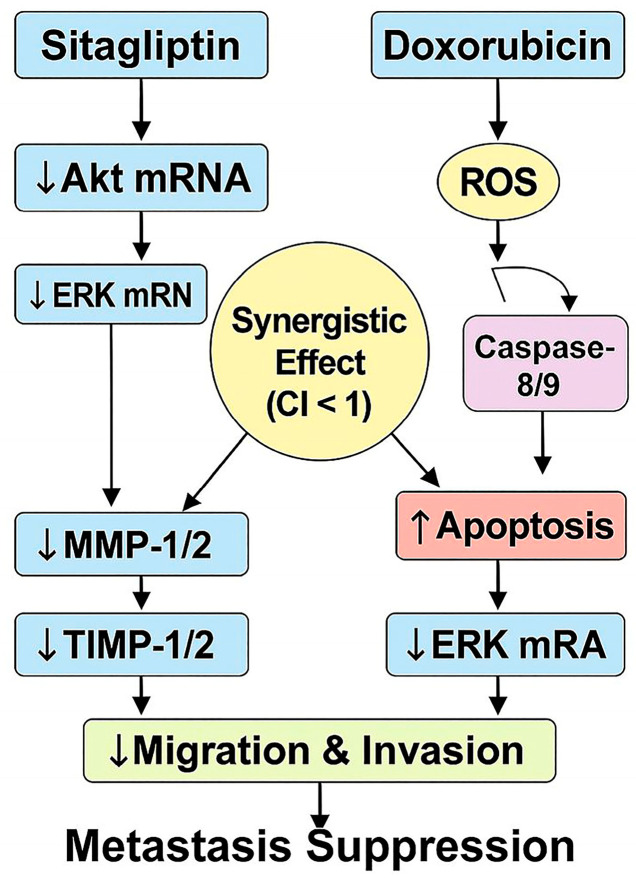
Proposed mechanistic model of the synergistic anticancer effects of sitagliptin and doxorubicin in HeLa cells. Sitagliptin treatment leads to transcriptional downregulation of Akt and ERK mRNA, resulting in decreased expression of MMP-1/2 and TIMP-1/2, which collectively suppress cell migration and invasion. Doxorubicin induces reactive oxygen species (ROS) generation, triggering caspase-8/9 activation and subsequent apoptosis. In parallel, doxorubicin also contributes to ERK mRNA downregulation, consistent with qRT-PCR findings. The combination of sitagliptin and doxorubicin produces a synergistic anticancer effect (CI < 1), amplifying apoptotic signaling and anti-metastatic outcomes. This model is based on transcriptional and functional data obtained in the present study and illustrates the integrated pathways underlying migration/invasion inhibition and metastasis suppression.

Together, these coordinated effects reveal a comprehensive mechanism by which sitagliptin and doxorubicin jointly promote apoptosis, disrupt pro-metastatic protease activity, and inhibit key survival pathways in cervical cancer cells. Figure 13 provides an integrated mechanistic overview, illustrating how sitagliptin-mediated Akt/ERK suppression and doxorubicin-induced oxidative stress converge to produce a synergistic anticancer response with enhanced apoptosis and reduced metastatic potential.

Together, these results support the potential of sitagliptin as an adjuvant agent capable of potentiating doxorubicin efficacy in cervical cancer therapy. Nevertheless, further in vivo validation and mechanistic exploration are essential to determine the translational feasibility and therapeutic applicability of this combination in clinical settings.

## Figures and Tables

**Table 1:** qRT-PCR primer sequences used for Akt, ERK, and GAPDH genes.

Gene	Forward Primer (5′→3′)	Reverse Primer (5′→3′)	Amplicon Size (bp)
Akt (AKT1)	AGCGACGTGGCTATTGTGAAG	GCCATCATTCTTGAGGAGGAAGT	124 bp
ERK1 (MAPK3)	AGCAGCCTCCTGATGACTTTG	TGGAGGTTGCTGAGGTGTTG	132 bp
ERK2 (MAPK1)	TGGCAAGAGATGTGGTGTGAA	CGTAGGTCAGCGTCTTGATG	119 bp
GAPDH	GAAGGTGAAGGTCGGAGTC	GAAGATGGTGATGGGATTTC	138 bp

**Table 2:** Predicted and overlapping molecular targets identified for sitagliptin and doxorubicin based on PubChem, SwissTargetPrediction, DrugBank, STRING, and GeneMANIA analyses.

Category	Targets
PI3K/Akt pathway	AKT1
MAPK/ERK pathway	MAPK1 (ERK2), MAPK3 (ERK1)
Apoptosis regulators	CASP8, CASP9, BAX, BCL2
ECM remodeling proteins	MMP-1, MMP-2, TIMP-1, TIMP-2
Oxidative stress signaling	NFE2L2 (Nrf2)

## Data Availability

All experimental data generated or analyzed during this study are available from the corresponding author upon request.
